# Supplementary material for: RNA-Seq Transcriptome Analysis of Differentiated Human Oligodendrocytic MO3.13 Cells Shows Upregulation of Genes Involved in Myogenesis
Source: Int J Mol Sci. 2022 May 25;23(11):5969. doi: 10.3390/ijms23115969 (PMC9181232; doi:10.3390/ijms23115969)
Supplement: Supplementary file 1 [file ijms-23-05969-s001.zip › ijms-1678890-supplementary/ijms-1678890-supplementary.pdf]

RNA-seq transcriptome analysis of differentiated human oligodendrocytic MO3.13 cells shows up-regulation of genes involved in myogenesis

Aleksandra Głowacka[1], Ewa Kilanczyk[2], Małgorzata Maksymowicz[1], Małgorzata Zawadzka[1]
Wiesława Leśniak[1], Anna Filipek[1]✉

[1]Nencki Institute of Experimental Biology, Polish Academy of Sciences, 3 Pasteur Street, 02-093 Warsaw, Poland
[2]Department of Medical Biology, Pomeranian Medical University, 1 Rybacka Street, 70-204 Szczecin, Poland

✉**Corresponding author:**
Anna Filipek
Nencki Institute of Experimental Biology, Polish Academy of Sciences,
3 Pasteur Street, 02-093 Warsaw, Poland,
e-mail: a.filipek@nencki.edu.pl

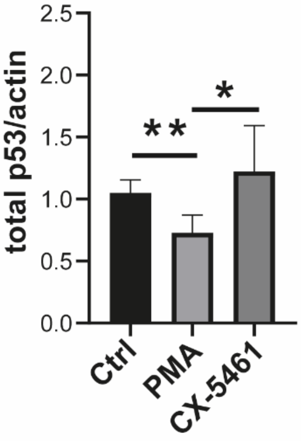

Figure S1. The level of total p53 in control (Ctrl), PMA or CX-5461 MO3.13 treated cells. Results are presented as a ratio of total p53 normalized to actin (loading control). Densitometric analysis of results (n=6) was performed with the use of one sample *t* test. Results are presented as means ± standard error**.** The level of statistical significance is indicated using $*p \leq 0.05$, $**p \leq 0.01$.

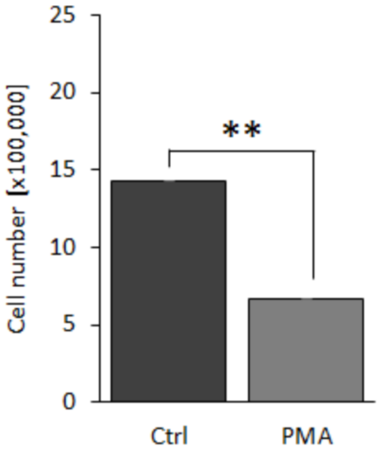

Figure S2. Analysis of cell proliferation. MO3.13 cells were cultured for 72 h in the absence (control; Ctrl) or presence of PMA (n=4), $^{**}p \leq 0.01$.

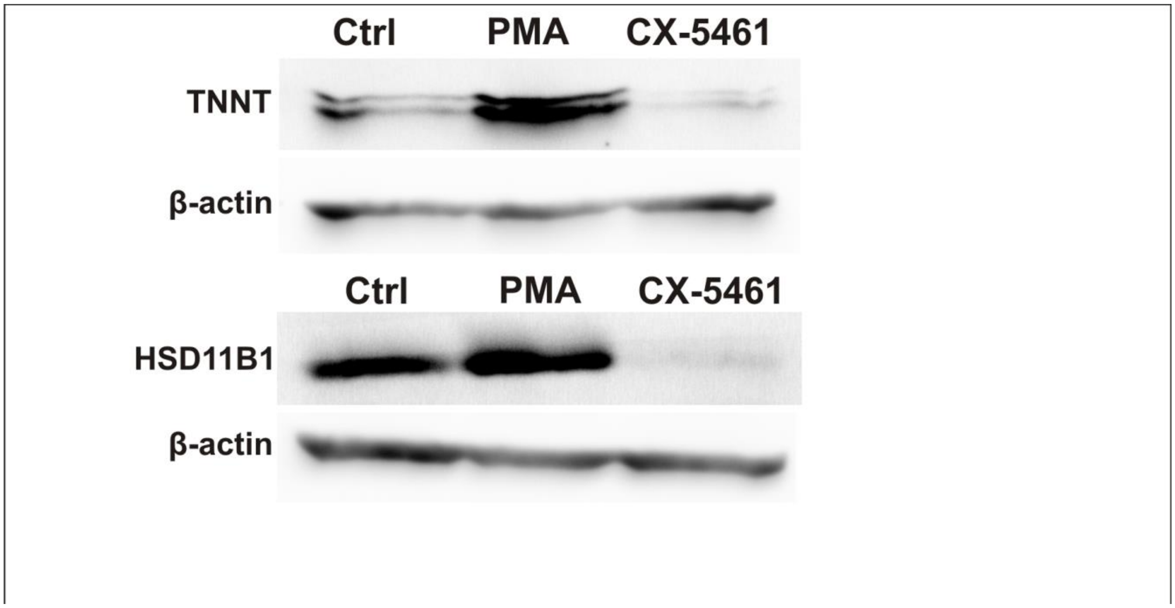

Figure S3. The protein level of TNNT and HSD11B1 in MO3.13 cells, control (Ctrl) and treated with PMA or CX-5461. Actin served as loading control.

Table S1. Sequences of primers used in RT-qPCR.

Gene	Forward Primer (5'->3')	Reverse Primer (5'->3')
18S	CGCCGCTAGAGGTGAAATTC	TTGGCAAATGCTTTCGCTC
CASQ2	AGCTTGTGGAGTTTGTGAAG	GGATTGTCAGTGTTGTCCC
HSD11B1	GACAGCGAGGTCAAAAGAAA	GTCCTCCCATGAGCTTTCCTG
*MYH **	GAGGACATGGCCATGATGAC	GGCATTGTCAGAGATGGAGAAG
TNNT3	AGGAGGAGGATGCCAAGAGGA	TCCTTGGCCTTGTCCCTCAGTTT
GPR17	GAGAGATGCTGAAACTCTCAGC	CAGGGAGAAGTTGGTGATCAGAC
MBP	ACCCAAGATGAAAACCCCGTA	TCCGTAGCCAAATCCTGGTCT
MOG	TTTTGATCCCCACTTTCTGAGG	CGTAGCTCTTCAAGGAATTGCC

Table S2. MO3.13 genes with statistically significant change in expression after PMA treatment. 6769 genes were up-regulated and 5856 - down-regulated.

（Table S2, please see the excel file）
